# A novel oxidative-stress related lncRNA signature predicts the prognosis of clear cell renal cell carcinoma

**DOI:** 10.1038/s41598-023-32891-z

**Published:** 2023-04-07

**Authors:** Yu Zhang, Guozhong Zhou, Wei Shi, Weili Shi, Meijun Hu, Defu Kong, Rong Long, Nan Chen

**Affiliations:** 1 Department of Endocrinology, Anning First People’s Hospital Affiliated to Kunming University of Science and Technology, Kunming, 650302 Yunnan China; 2 Department of Science and Research, Anning First People’s Hospital Affiliated to Kunming University of Science and Technology, Kunming, 650302 Yunnan China

**Keywords:** Nephrology, Oncology, Cancer, Cancer genomics, Cancer models

## Abstract

Clear cell renal cell carcinoma (ccRCC) is a primary malignant tumour of tubular epithelial origin and is most common in the urinary tract. Growing evidence suggests that oxidative stress (OS), generates high levels of reactive oxygen species (ROS) and free radicals, and plays a critical role in cancer in humans. However, the predictive value of OS-related long non-coding RNAs (lncRNAs) in ccRCC remains unclear. We constructed a predictive signature of survival based on OS-related lncRNAs that were obtained from The Cancer Genome Atlas (TCGA–KIRC), to predict the prognosis of patients with ccRCC. The signature comprised seven lncRNAs: SPART-AS1, AL162586.1, LINC00944, LINC01550, HOXB-AS4, LINC02027, and DOCK9-DT. OS-related signature of lncRNAs had diagnostic efficiency higher than that of clinicopathological variables, with an area of 0.794 under the receiver operating characteristic curve. Additionally, the nomogram based on risk scores and clinicopathological variables (age, gender, grade, stage, M-stage, and N-stage) showed strong predictive performance. Patients with high-risk were found to be more sensitive to the therapeutic drugs ABT.888, AICAR, MS.275, sunitinib, AZD.2281, and GDC.0449. Our constructed the predictive signature can independently predict the prognosis of patients with ccRCC; however, the underlying mechanism needs further investigation.

## Introduction

Renal cell carcinoma is a common form of kidney cancer, and accounts for approximately 80–90% malignant kidney tumours^1,2^. Among its various subtypes, clear cell renal cell carcinoma (ccRCC) is most common, accounting for approximately 85% renal cell carcinomas and causing 14,000 deaths annually in the US^3–5^. Disease symptoms can be improved by targeting the vascular endothelial growth factor pathway using anti-angiogenic agents including the tyrosine kinase inhibitors sunitinib and pazopanib; however, overall and progression-free survivals remain low in patients with ccRCC, and some patients develop recurrent metastases after nephrectomy, which poses a significant medical burden to the society^5,6^. Therefore, exploring new potential markers for prognosis and individualised treatment is necessary.

Oxidative stress (OS) occurs owing to an imbalance between oxidative and antioxidant activities in the body, and leads to inflammatory infiltration by neutrophils, increased protease secretion, and production of large amounts of oxidative intermediates. OS damages DNA molecules, proteins, and lipids; alters signalling pathways; and regulates the progression of various cancers^7–10^. Cancer cells typically have higher levels of reactive oxygen species (ROS) than normal cells. In the presence of sustained OS, cancer cells may have evolved a set of specific adaptive mechanisms, which potentiates their ROS-scavenging system to deal with OS and inhibit apoptosis and contribute to the development of cancer^6,11,12^. Metabolomic analyses have shown that metabolites in the glycolytic pathway are abundant more than two-fold in ccRCC than those in normal kidneys; biomolecules related to the metabolism of the ROS scavenger glutathione, including cysteine and gamma-glutamyl cysteine, are elevated in advanced ccRCC and associated with poor survival outcomes in patients with ccRCC^13^.

Long non-coding RNAs (lncRNAs) contain more than 200 nucleotides. They are major regulators of gene expression and play key roles in various biological functions and disease processes, including cancer^14^. LncRNAs contribute to energy metabolism and cancer progression through post-translational modification of key metabolism-related proteins via ubiquitination, phosphorylation, and acetylation^15–17^. In 2016, a systematic evaluation and meta-analysis (including 111 studies on the prognostic impact of lncRNAs on malignancies) identified the impact of lncRNAs on overall patient survival in 83% of the studies, confirming that multiple lncRNAs are significantly associated with the prognosis of cancers^18^.

We hypothesise that OS plays a key role in ccRCC. Therefore, this study aimed to construct novel survival signature based on differentially expressed OS-related lncRNAs as obtained from The Cancer Genome Atlas (TCGA), thereby providing a reliable prognostic signature.

## Results

### Screening and enrichment of differential genes

**Figure 1:**
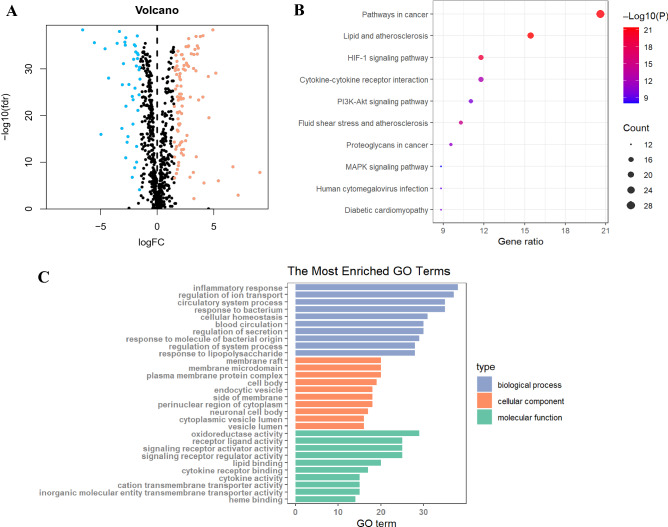
GO and KEGG analyses of OS-related DEGs in cancer and adjacent tissues. (**A**) Volcano plot of 137 OS-related genes in ccRCC. Red and blue dots represent up-regulated and down-regulated genes, respectively. (**B**) KEGG analysis of OS-related DEGs. (**C**) GO analysis of OS-related DEGs. GO, Gene Ontology; KEGG, Kyoto Encyclopaedia of Genes and Genomes; DEGs, differentially expressed genes; FC, fold change; FDR, false discovery rate; BP, biological process; CC, cellular components; MF, molecular function.

In this study, we used the data of 507 patients with ccRCC from TCGA cohort (tumour group, 435; normal group, 72). We identified 136 differentially expressed genes (DEGs) associated with OS, including 95 up-regulated genes and 41 down-regulated genes (Fig. 1A). Kyoto Encyclopaedia of Genes and Genomes (KEGG) pathway analysis showed that OS-related DEGs were mainly enriched in pathways associated with cancer, lipid and atherosclerosis, hypoxia-inducible factor 1 (HIF-1) signalling pathway, cytokine-cytokine receptor interactions, PI3K-Akt signalling pathway, proteoglycan metabolism in cancer, infection of human cytomegalovirus, diabetic cardiomyopathy, and mitogen-activated protein kinase (MAPK) signalling pathway (Fig. 1B). Gene Ontology (GO) analysis showed that OS-related DEGs were mainly enriched in (a) biological processes related to inflammatory responses, regulation of ion transport, and processes of the circulatory system; (b) cellular composition related to membrane raft, membrane microdomain, and plasma membrane protein complex; and (c) molecular functions related to the oxidoreductase activity, receptor and ligand activity, and signalling receptor activator activity. (Fig. 1C).

### Construction of a predictive signature for OS-related lncRNAs

**Figure 2:**
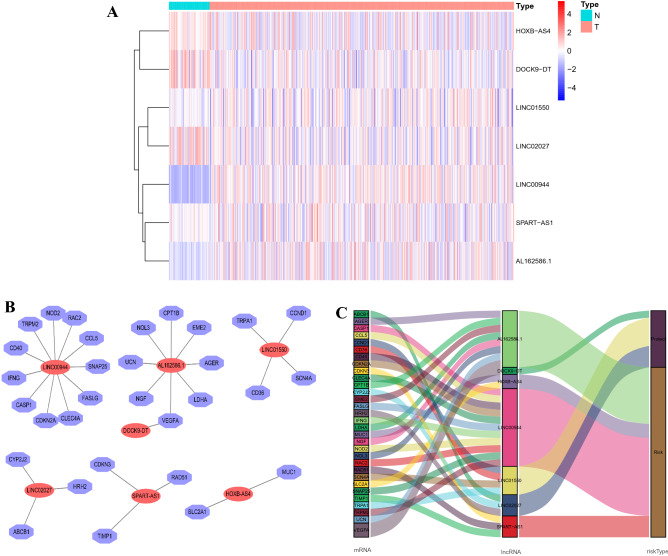
Expression and lncRNA–mRNA network of seven OS-related lncRNAs in the predictive signature. (**A**) Expression of seven OS-related lncRNAs in ccRCC and normal tissues. (**B**) The co-expression network of prognostic OS-related lncRNAs. (**C**) Sankey diagram of prognostic OS-based lncRNAs. lncRNAs, long non-coding RNAs; ccRCC, clear cell renal cell carcinoma; N, normal; T, tumour.

We filtered 1479 OS-related lncRNAs. Univariate Cox regression analysis showed that 274 lncRNAs were associated with the prognosis of patients with ccRCC. Multifactorial Cox regression analysis revealed that seven OS-associated lncRNAs, including SPART-AS1, AL162586.1, LINC00944, LINC01550, HOXB-AS4, LINC02027, and DOCK9-DT, constructed a predictive signature. The expression of these seven lncRNAs is shown in Fig. 2A.

We used Cytoscape to visualise the predictive lncRNAs. In terms of co-expression with OS-related genes, LINC00944 co-expressed with eleven genes (*CD40*, *TRPM2*, *NOD2*, *RAC2*, *CCL5*, *SNAP25*, *FASLG*, *CLEC4A*, *CDKN2A*, *CASP1,* and *IFNG*); AL162586.1 with eight genes (*UCN*, *NOL3*, *CPT1B*, *EME2*, *AGER*, *LDHA*, *VEGFA,* and *NGF*); LINC01550 with four genes (*TRPA1*, *CCND1*, *SCN4A,* and *CD36*); LINC02027 with three genes (*CYP2J2*, *HRH2,* and *ABCB1*); SPART-AS1 with three genes (*CNKN3*, *RAD51,* and *TIMP1*); HOXB-AS4 with two genes (*SLC2A1* and *MUC1*); and DOCK9-DT with one gene (*VEGFA*) (Fig. 2B).

DOCK9-DT, LINC01550, and LINC02027 were protective factors, whereas HOXB-AS4, SPART-AS1, AL162586.1, and LINC00944 constituted risk factors (Fig. 2C). Risk scores were calculated as follows:

$$\mathrm{Risk score }= (-1.101 \times \mathrm{ DOCK}9-\mathrm{DT expression}) + (-0.706 \times \mathrm{ LINC}01550\mathrm{ expression}) + (-0.433 \times \mathrm{ LINC}02027\mathrm{ expression}) + (0.283 \times \mathrm{ HOXB}-\mathrm{AS}4\mathrm{ expression}) + (0.447 \times \mathrm{ SPART}-\mathrm{AS}1\mathrm{ expression}) + (0.689 \times \mathrm{ AL}162586.1\mathrm{ expression}) + (0.690 \times \mathrm{ LINC}00944\mathrm{ expression}).$$

### Correlation between the predictive signature and prognosis of patients with ccRCC

**Figure 3:**
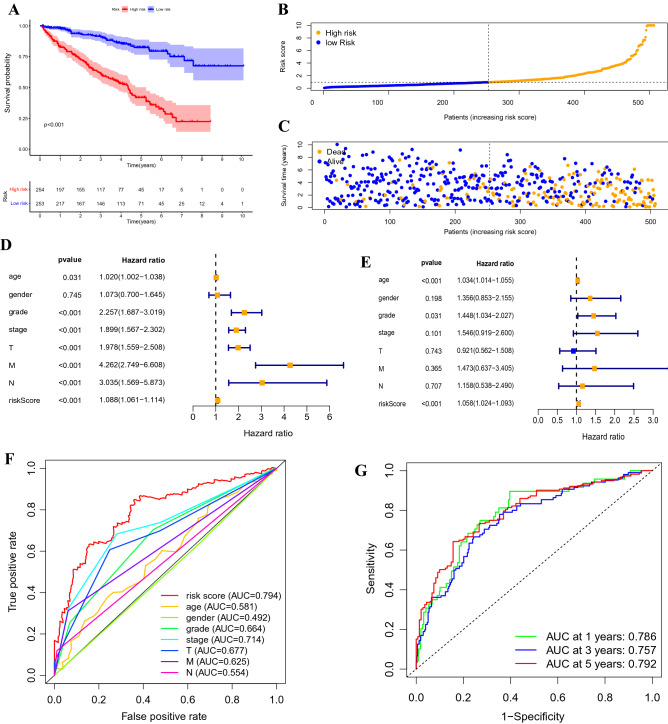
Correlation between the predictive signature and prognosis of patients with ccRCC. (**A**) Kaplan–Meier analysis of the overall survival rate of patients with ccRCC in the high-risk and low-risk groups. (**B**) The distribution of risk score among patients with ccRCC. (**C**) The number of dead and alive patients with different risk scores. Blue and yellow represents the numbers of survivors and deaths, respectively. (**D**) Forest plot for univariate Cox regression analysis. (**E**) Forest plot for multivariate Cox regression analysis. (**F**) The ROC curve of the risk score and clinicopathological variables. (**G**) ROC curve and AUCs at 1, 3, and 5 years survival for the predictive signature. ccRCC, clear cell renal cell carcinoma; ROC, receiver operating characteristic; AUC, area under the curve; T, tumour; N, lymph node.

Risk scores were calculated for all patients, who were then divided into high-risk (n = 254) and low-risk (n = 253) groups based on the median risk score. According to the description of Kaplan–Meier survival curve, the overall survival was significantly lower in the high-risk group than that in the low-risk group (Fig. 3A, p < 0.001). The distribution of risk scores displayed comparatively high scores in the high-risk group (Fig. 3B). The scatter plot indicated that the survival time in the high-risk group was lower than that in the low-risk group (Fig. 3C), with 5-years survival rates of 17.7% and 28.1% for the high-risk and low-risk groups, respectively.

Univariate Cox regression analysis showed that age, grade, stage, T-stage, M-stage, N-stage, and risk score were significantly associated with overall survival in patients with ccRCC (Fig. 3D). Multiple Cox regression analysis showed that age, grade, and risk score were independent predictors of overall survival of patients (Fig. 3E). The area under the curve (AUC) value for the risk score was 0.794, which was better than those of all clinicopathological variables for predicting the prognosis of patients with ccRCC (Fig. 3F). The AUC values of 1-, 3-, and 5-years survival were 0.786, 0.757, and 0.792, respectively, indicating good predictive performance (Fig. 3G).

**Figure 4:**
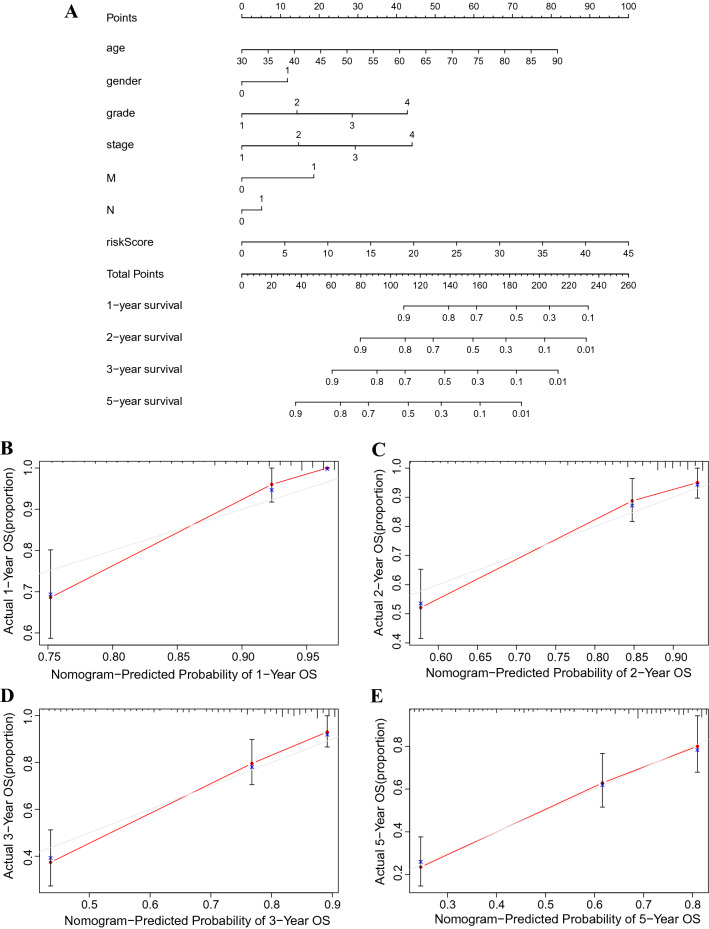
Construction and verification of the nomogram. (**A**) Nomogram combining clinicopathological variables and risk scores to predict overall survival at 1, 2, 3, and 5 years of patients with ccRCC. (**B**–**E**) The calibration curves testing the agreement of actual overall survival rates with predicted survival at 1, 2, 3 and 5 years. N, lymph nodes; ccRCC, clear cell renal cell carcinoma.

To further predict the prognosis of patients with ccRCC, we constructed a nomogram containing clinicopathological variables and risk scores to predict the prognosis of patients with ccRCC at 1, 2, 3, and 5 years (Fig. 4A). The calibration curve showed excellent agreement between the actual overall survival rates and predicted survival rates for 1, 2, 3, and 5 years (Fig. 4B–E).

### The relationship between predictive signature and prognosis of patients with ccRCC under different clinicopathological variables

**Figure 5:**
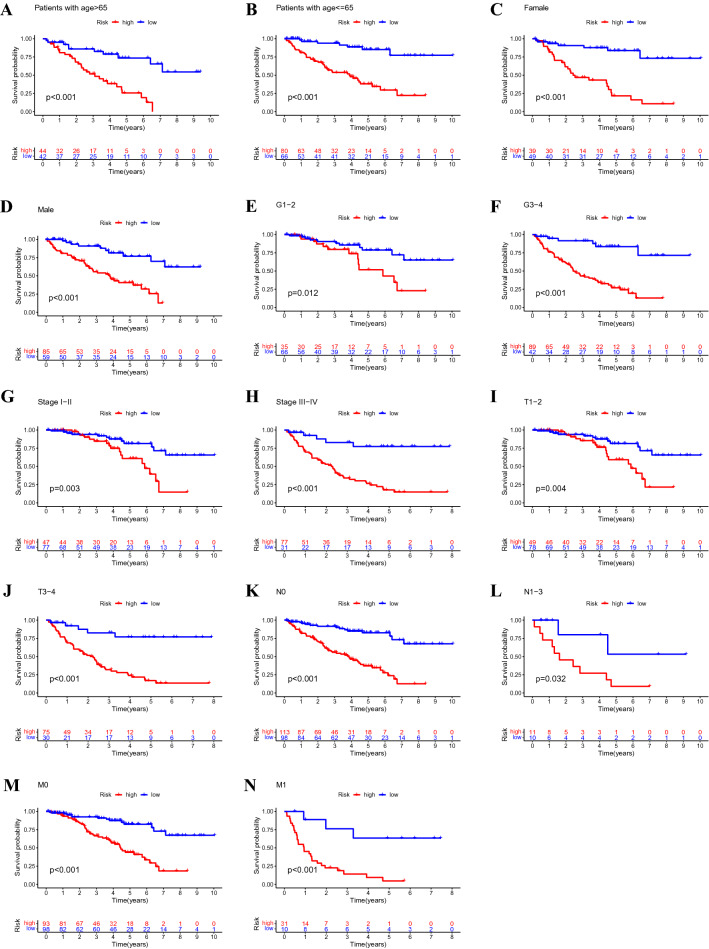
Kaplan–Meier survival curves for high-risk and low-risk groups in patients divided according to different clinicopathological variable classifications. (**A**,**B**) Age. (**C**,**D**) Gender. (**E**,**F**) Grade. (**G**,**H**) Stage. (**I**,**J**) T stage. (**K**,**L**) N stage. (**M**,**N**) M stage. T, tumour; N, lymph node; M, metastasis.

All patients with ccRCC were divided into groups according to age, gender, grade, cancer stage, T-stage, N-stage, and M-stage. For each classification, patients in the high-risk group had significantly shorter overall survival than those in the low-risk group (Fig. 5). These results suggest that the predictive signature can predict the prognosis of patients with ccRCC without considering clinicopathological variables.

### Internal validation of predictive signature

**Figure 6:**
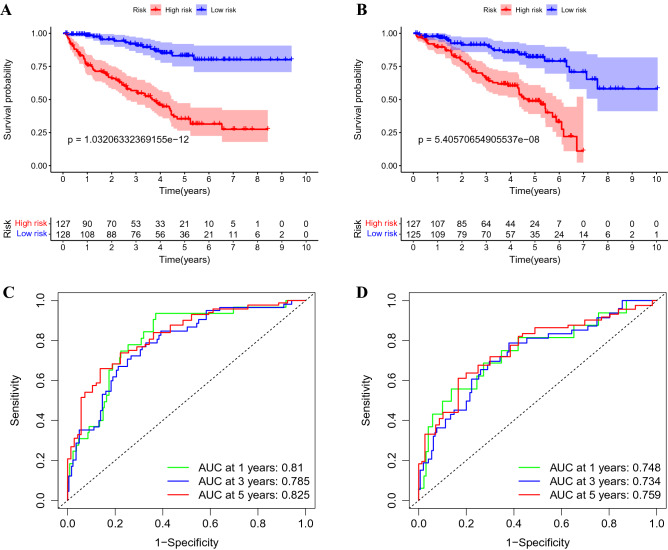
Internal validation of overall survival prediction signature based on the entire TCGA dataset. (**A**) Kaplan–Meier survival curves for the training cohort. (**B**) Kaplan–Meier survival curves for the testing cohort. (**C**) ROC curves and AUC values for 1-, 3-, and 5-years survival for the training cohort. (**D**) ROC curves and AUC values for 1-, 3-, and 5-years survival for the testing cohort. TCGA, the Cancer Genome Atlas; ROC, receiver operating characteristic; AUC, area under the curve.

To confirm the reliability of the predictive signature, we divided 507 randomly selected predictive signature into two groups: 255 patients for training set and 252 for testing set. In both sets, patients in the high-risk group had lower overall survival rates than those in the low-risk group (Fig. 6A,B, p < 0.05), and the receiver operating characteristic (ROC) curves for both groups showed good predictive performance. In the training set, the AUC values of 1-year, 3-years, and 5-years survival were 0.81, 0.785, and 0.825, respectively (Fig. 6C), and those in the testing set, were 0.748, 0.734, and 0.759, respectively (Fig. 6D).

### Immune infiltration and its pathway analysis

**Figure 7:**
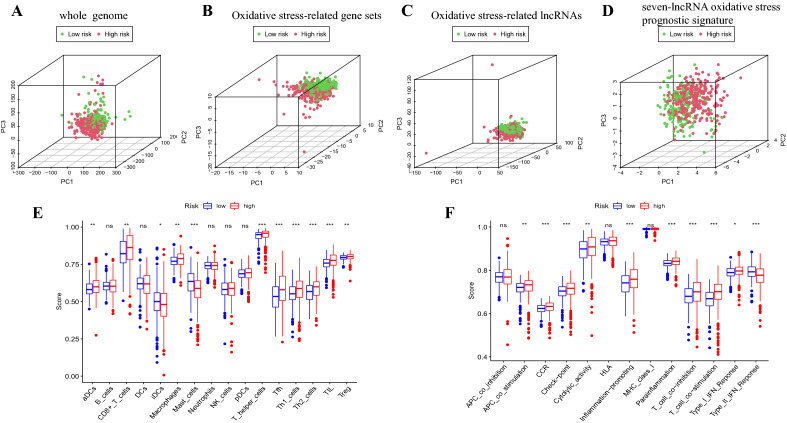
Patients with high and low risk scores have different immune status and scores for immune infiltrating cells and immune-related functions in the high-risk and low-risk groups. (**A**) Genome-wide distribution of patients. (**B**) OS-related gene sets. (**C**) OS-related lncRNAs. (**D**) Seven-lncRNA prognostic signature of OS. (**E**) Infiltration levels of 16 immune cells in the high-risk and low-risk groups using the ssGSEA algorithm. (**F**) Correlation of predictive signature with 13 immune-related functions. OS, oxidative stress; lncRNA, long non-coding RNA; ssGSEA, single-sample gene set enrichment analysis; aDCs, activated dendritic cells; iDCs, immature dendritic cells; NK, natural killer; pDCs, plasmacytoid dendritic cells; Tfh, T follicular helper cells; Th1, T helper cell type 1; TIL, tumour-infiltrating lymphocytes; Treg, regulatory T cell; APC, antigen presenting cell; CCR, cytokine–cytokine receptor interaction; HLA, human leukocyte antigen; MHC, major histocompatibility complex; IFN, interferon. *p < 0.05; **p < 0.01; ***p < 0.001; *ns,* not significant.

We used principal component analysis maps to visualise the distribution of patients based on the whole genome, OS-associated genome, OS-associated lncRNAs, and the seven lncRNAs identified as prognostic signature of OS. Patients with high-risk and low-risk scores in the lncRNA signature group were clearly distributed in different quadrants, indicating that this is the best way to differentiate among patients (Fig. 7A–D). Owing to differential prognoses of patients in the high-risk and low-risk groups, we performed gene set enrichment analysis (GSEA). Pathways for primary immunodeficiency, intestinal immune network for immunoglobulin A production, and cytokine–receptor interaction were significantly enriched in the high-risk group (Table 1), suggesting that high risk is closely associated with immune responses.

**Table 1:** The gene sets enriched in high-risk group.

Gene set	ES	NES	NOM *p*-val	FDR *q*-val
Primary immunodeficiency	0.61	1.79	0.060	0.480
Homologous recombination	0.61	1.78	0.023	0.248
Hematopoietic cell lineage	0.44	1.72	0.038	0.241
Intestinal immune network for IGA production	0.55	1.71	0.04	0.20
Cytokine receptor interaction	0.36	1.67	0.039	0.165

To explore the correlation between risk scores and immune cells and functions in patients with ccRCC, we quantified the single-sample gene set enrichment analysis (ssGSEA) for subpopulations of different immune cells, related functions and pathways. The results showed that the levels of infiltration by activated dendritic cells (aDCs), CD8(+) T cells, immature dendritic cells (iDCs), macrophages, mast cells, T helper cells, T follicular helper (Tfh) cells, T helper type 1 (Th1) cells, T helper type 2 (Th2) cells, tumour-infiltrating lymphocytes (TIL), and regulatory T cell (Treg) were significantly different between the high-risk and low-risk groups (Fig. 7E).

Co-stimulation of antigen-presenting cell, cytokine–cytokine receptor interaction (CCR), chemokine receptor checkpoint, cytolytic activity, promotion of inflammation, para-inflammation, co-inhibition and co-stimulation of T cells, and response of type I and type II interferons were higher in the high-risk group than those in the low-risk group (Fig. 7F). These findings also suggest that immune function is more active in the high-risk group than that in the low-risk group.

### Correlation between predictive signature and treatment of ccRCC

**Figure 8:**
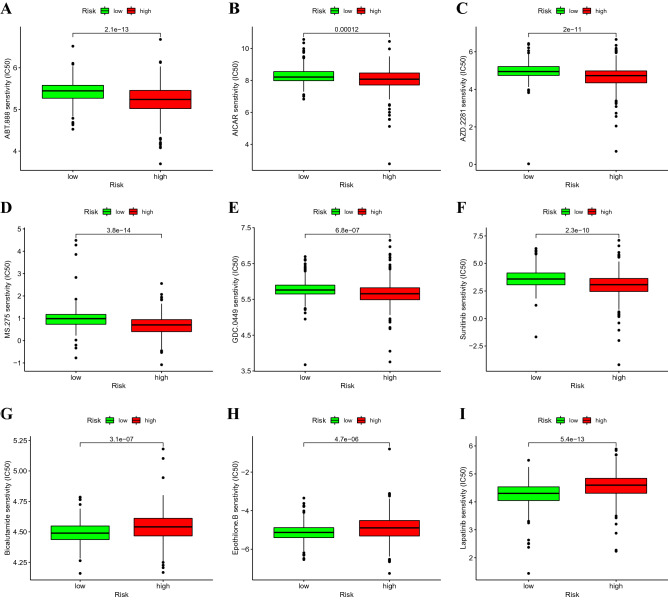
Comparison of drug sensitivity between high-risk and low-risk groups. The half-maximal inhibitory concentrations (IC_50_) of (**A**) ABT.888, (**B**) AICAR, (**C**) AZD.2281, (**D**) MS.275, (**E**) GDC.0449, (**F**) sunitinib, (**G**) bicalutamide, (**H**) epothilone B, and (**I**) lapatinib in high-risk and low-risk groups.

To further explore individualised treatment regimens, we compared the estimated half-maximal inhibitory concentrations (IC_50_) of 85 chemotherapeutic drugs or inhibitors between high-risk and low-risk groups. We noticed that ABT.888, AICAR, MS.275, sunitinib, AZD.2281, and GDC.0449 may be used as candidate drugs for treating patients in the high-risk group. In contrast, bicalutamide, epothilone B, and lapatinib may not be ideal for patients in the high-risk group (Fig. 8).

## Discussion

As the most common type of renal cell carcinoma, ccRCC originates from the proximal convoluted tubules and has a recurrence rate of up to 40%, even after the treatment of local tumours^19^. It leads to high mortality owing to its aggressive and metastatic nature^20,21^. Recent studies have constructed signatures with m6, metastasis, glycolysis and immune-related lncRNAs to predict the prognosis of patients with ccRCC^22–25^. However, OS-related lncRNA signatures have not been constructed.

We identified 136 DEGs associated with OS, and KEGG enrichment analysis revealed the pathways, including the HIF-1 and MAPK signalling pathways, in which these genes were mainly enriched. The inflammatory signalling pathways of nuclear factor kappa B and cyclooxygenase 2 (as mediated by interleukin-1β) up-regulate the HIF-1α pathway that up-regulates the expression of vascular endothelial growth factor, which is required for effective angiogenesis for subsequent tumour growth and metastasis^26^. Additionally, MAPK signalling is a well-defined pathway in cancer biology, and its overactivation causes more than 40% of human cancers^27^.

We identified seven differentially expressed lncRNAs, including SPART-AS1, AL162586.1, LINC00944, LINC01550, HOXB-AS4, LINC02027, and DOCK9-DT, which can act as independent prognostic signature for ccRCC. Previously, LINC00944 has been validated as a cancer-associated lncRNA^28^; it is highly expressed in renal cell carcinoma tissues, and its high expression is associated with tumour stage, tumour-infiltrating T lymphocytes, and pro-apoptotic markers^29,30^. LINC01550 is significantly down-regulated in melanoma tissue, and up-regulation of LINC01550 significantly inhibits the proliferation and invasive capacity of melanoma cells along with induced apoptosis and arrest of the G1 and S phases^31^. LINC02027 and HOXB-AS4 are important prognostic signatures for ccRCC^32–34^; DOCK9-DT is important in thyroid cancer^35,36^; and AL162586.1 is important for bladder cancer^37^. We calculated risk scores and confirmed that the survival of patients with ccRCC was significantly lower in a high-risk group than that in the low-risk group, with ROC curves indicating that prognostic features based on the identified lncRNAs had good predictive performance. Our results identify efficient and significant prognostic ability for ccRCC using OS-related lncRNA signature.

Analyses of immune infiltration showed large numbers of aDCs, CD8(+) T cells, iDCs, macrophages, mast cells, T helper cells, Tfh cells, Th1 cells, Th2 cells, TIL and Tregs in the high-risk group. Kidney tissue from late-stage ccRCC tumours has been found to be highly infiltrated by CD8(+) T cells^38^. Additionally, Tregs, Th2 cells, and macrophages positively correlate with the proliferation of renal cancer cells and invasion of renal A498 and Caki-1 cells^39^. We also found that patients in the high-risk group were sensitive to ABT.888, AICAR, MS.275, sunitinib, AZD.2281, and GDC.0449. AT.888 has been used for treating a wide range of tumours^40,41^. AICAR, a widely used adenosine monophosphate protein kinase (AMPK) activator, protects against cisplatin-induced kidney injury^42^. However, patients at high-risk are resistant to drugs such as bicalutamide, epothilone B, and lapatinib. Therefore, the present study can be utilised to provide personalised and precise drug treatments for patients with high-risk.

Our study has several limitations. First, we used the TCGA database only and did not validate the study using other clinical datasets. Second, because the data were downloaded from TCGA, a large proportion of unknown lncRNAs were missing, providing relatively limited raw data for initial analysis. Our inability to obtain a complete dataset that simultaneously includes information on lncRNA expression, clinicopathological characteristics, and survival outcomes of patients with ccRCC may have influenced the construction of this signature. Finally, we did not determine the specific mechanisms linking the OS-related lncRNA signature to the ccRCC prognosis and efficacy of drug treatment in patients with ccRCC. Further experimental validation with a large patient population is needed.

In conclusion, we constructed a new signature of OS-related lncRNAs, which can independently predict the prognosis of patients with ccRCC. This study provides a basis and potential predictive significance for the possible mechanisms linking OS-related lncRNAs with ccRCC and may help in developing new clinical interventions.

## Methods

### Data download

We downloaded the fragments per kilobase of transcript per million mapped reads (FPKM)-normalised transcript RNA-sequencing data from TCGA website (https://portal.gdc.cancer.gov) and corresponding clinical and prognostic data from the TCGA ccRCC dataset; the latter contained data for 507 patients, including lncRNA expression values and survival times. OS-related genes were extracted from GeneCards (https://www.genecards.org) with a relevance score ≥ 3.

### Functional enrichment analysis of DEGs related to OS

We used a false discovery rate (FDR) < 0.01 and |log 2-fold change| > 1.5 as screening criteria to identify DEGs associated with OS. The genes were analysed using Metascape (https://metascape.org)^43^ for GO and KEGG analysis and were visualised using ggplot2.

### Construction of a predictive signature for OS-related lncRNAs

We used limma to assess the correlation between OS-related genes and lncRNAs. Correlation coefficients |*R*^2^| > 0.3 and *p* < 0.001 were used as screening criteria. We analysed the screened lncRNAs using univariate Cox regression to obtain lncRNAs associated with the prognosis of OS in patients with ccRCC. Multivariate Cox regression analysis was utilised to obtain OS-related lncRNAs that provide a predictive signature.

### Construction of a nomogram

We combined risk scores with the clinicopathological characteristics of age, gender, grade, stage, M-stage, and N-stage to construct a nomogram that predicted survival at 1, 2, 3, and 5 years in patients with ccRCC. Calibration curves were used to test whether predicted survival was consistent with the actual survival.

### Functional enrichment analysis of the lncRNA signature and immune infiltration analysis

Patients with ccRCC were divided into high-risk and low-risk groups based on their median risk score. Elevated and decreased lncRNAs were separately enriched using GSEA v4.1.0 (http://www.broad.mit.edu/gsea), with *p* < 0.05 and FDR < 0.25 as thresholds for statistical significance. The infiltration score of 16 immune cells and the activity of 13 immune-related pathways were calculated by ssGSEA using the GSVA software package.

### The predictive role of our signature related to clinical therapeutics

To assess the predictive role of the signature in relation to clinical response to treatment, we calculated the IC_50_ of commonly used chemotherapeutic agents for clinical treatment of ccRCC. The Wilcoxon signed-rank test was used to compare the IC_50_ values between the high-risk and low-risk groups.

### Statistical analysis

The Wilcoxon test was used to analyse expression of DEGs associated with OS in cancerous and normal tissues. Univariate Cox regression analysis was employed to analyse the relationship between lncRNAs associated with OS and overall survival. Multifactorial Cox regression analysis was applied to screen lncRNAs associated with OS to construct predictive signature. The Kaplan–Meier method and log-rank tests were used to analyse the overall survival of patients in high-risk and low-risk groups. The survival ROC package was used to plot the ROC curve of subjects and to determine AUC values. All statistical analyses were performed using R software v4.1.3 and Metascape.

## Data Availability

Some or all data, models, or code generated or used in the study are available from the corresponding author upon request.
